# Is Older Age Associated with Higher Self- and Other-Rated ASD Characteristics?

**DOI:** 10.1007/s10803-017-3444-2

**Published:** 2018-01-18

**Authors:** Anne G. Lever, Hilde M. Geurts

**Affiliations:** 1 Department of Psychology, Dutch Autism and ADHD Research Center, University of Amsterdam, Nieuwe Achtergracht 129B, 1018 WS Amsterdam, The Netherlands; 2 Dr. Leo Kannerhuis, Research Development & Innovation, Doorwerth, The Netherlands; 3 Department of Psychiatry, VU Medical Center, Amsterdam, The Netherlands; 4 Dimence, Deventer, The Netherlands

**Keywords:** Autism spectrum disorder, Self- and other-report, Autism traits, Aging, Adulthood, Symptomatology

## Abstract

Autism spectrum disorder (ASD) characteristics seem to abate over time, but whether this protracts until late adulthood is largely unknown. We cross-sectionally investigated self- and other-reported ASD characteristics of adults with (ASD: N_max-self_ = 237, N_max-other_ = 130) and without ASD (COM: N_max-self_ = 198, N_max-other_ = 148) aged 19–79 years. Within the ASD group, self-reported ASD characteristics, and sensory sensitivities were highest in middle adulthood, while age was not associated to empathy. Sex differences were also found. However, age-and sex-related differences were not revealed by others and self- and other-report were poorly concordant. These results show that ASD characteristics in adulthood are differently perceived across age, sex, and informants and suggest that it is important to repeatedly assess self-reported ASD characteristics during adulthood.

**Electronic supplementary material:**

The online version of this article (10.1007/s10803-017-3444-2) contains supplementary material, which is available to authorized users.

## Introduction

Although autism spectrum disorder (ASD) is considered a lifelong condition, there is evidence that behavioral ASD characteristics may abate over time (Magiati et al. 2014; Seltzer et al. 2004). For example, studies in children and young adults with ASD revealed that some might no longer meet ASD diagnostic criteria (Helles et al. 2016; Louwerse et al. 2015). This suggests that ASD characteristics and, potentially, the experienced associated impairments can actually change across the lifespan (Geurts and Jansen 2012; Happé et al. 2016; Howlin et al. 2013; Piven et al. 1996). Knowledge on behavioral ASD characteristics in middle and late adulthood is, however, still limited, even though critical in elucidating the magnitude and specificity of age-related differences and for recognizing ASD in (late) adulthood (e.g., Happé and Charlton 2012; Hategan et al. 2016; Perkins and Berkman 2012; Piven and Rabins 2011; Wright et al. 2013, 2016).

The few studies that do include mid- and/or old aged ASD adults present contradictory findings (e.g., Bastiaansen et al. 2011; Bishop and Seltzer 2012; Esbensen et al. 2009; Happé et al. 2016; Howlin et al. 2013; Shattuck et al. 2007). For example, it has been reported that ASD characteristics become less severe over time (e.g., Howlin et al. 2013), but also that older age was associated with higher ratings of ASD traits (e.g., Happé et al. 2016). There are a wide range of methodological differences (for example, respectively longitudinal, childhood ASD diagnoses, DSM-III criteria, and other-reports versus cross-sectional, adulthood ASD diagnoses, DSM-IV and DSM-V criteria, and self-reports) between these two studies which each could serve as valid explanations for the differences in observed findings. Furthermore, whether age-related differences in ASD characteristics are observed might depend on whether one focuses on specific ASD subdomains (e.g., Howlin et al. 2013) or on general ASD characteristics to get an overall picture (e.g., Happé et al. 2016).

For example, Shattuck et al. (2007) examined changes in other-reported ASD characteristics over a 4.5 years period among ASD individuals aged 10–52 years. Overall, while nonverbal communication impairments remained stable and symptoms of verbal communication and social reciprocity ameliorated, improvement was especially observed in the repetitive behavior domain. Similarly, other-reported ASD severity decreased over an approximately 37 years period (age range at follow-up 29–64 years), with, again, significant improvement on the restricted, repetitive behavior domain (Howlin et al. 2013). This is in line with the finding that older individuals with ASD (until 62 years) displayed fewer and less severe repetitive behaviors than younger individuals as reported by other informants (Esbensen et al. 2009). Regarding sensory sensitivity, newly relevant in the DSM-V (American Psychiatric Association 2013), self-reported sensory symptoms were not associated with age in the broad general population (range 16–65 years) (Robertson and Simmons 2013) nor in adults with ASD (18–65 years) (Crane et al. 2009). Anecdotal accounts also indicated that sensory symptoms do not seem to abate, although people might be better able to cope with them (Grandin 2011), which might explain why parents reported age-related improvements (Kern et al. 2006; Shattuck et al. 2007) contrary to self-report. Regarding social behavioral characteristics, older adults were socially more adjusted than younger adults (range 18–54 years) according to both self and another informant, even though age was not related to observed social symptoms (Bastiaansen et al. 2011).

Even studies with apparently similar methods may display contradictory findings. For example, age was (e.g., Happé et al. 2016) or was not (e.g., Bishop and Seltzer 2012; De la Marche et al. 2015) related to general self-reported ASD traits among adults. In a recent report of ASD adults, predominantly aged between 18 and 55 years, older age was associated with higher ratings of ASD traits including traits related to social symptomatology (Happé et al. 2016). While this observation might be explained by the recently obtained adulthood ASD diagnoses of the participants, the authors hypothesized that this could also be due to an age-related improvement in the insight in one’s own functioning. If this is indeed the case, a similar age-related association might be absent when other informants rate ASD-symptomatology. In sum, repetitive behaviors seem to mitigate with increasing age, whereas the findings of other ASD symptom domains are less consistent which might partly be informant related.

A common characteristic of most of the aforementioned ASD studies is that the number of participants over the age of 55 years was relatively small or non-existent. This is relevant as we recently showed that psychiatric comorbidity (Lever and Geurts 2016b) in adults with ASD differs between older individuals (aged 55–79 years) and slightly younger individuals with ASD (aged 39–54 years). The presence of psychiatric comorbidities was lower in the older than in the younger group, but almost similar to those that were much younger (aged 19–38 years). Moreover, in a general cross-sectional sample both cognitive and affective components of empathy increased from young to middle adulthood and declined in late adulthood (O’Brien et al. 2013). This is of relevance for ASD as ASD individuals are thought to have problems with cognitive empathy rather than affective empathy (Jones et al. 2010). These empathy difficulties are often related to the social challenges ASD individuals experience in daily life. Inverted U-shapes (i.e., an increase followed by a decrease) will be missed if hardly any older participants are included when determining lifespan ASD symptomatology.

As mentioned, inconsistencies between age-related findings could be related to who is the informant providing information. Direct comparisons of the ratings of different informants indicate that informants generally disagree on psychopathological measures (e.g., Achenbach et al. 2005; Kooij et al. 2008; Samuel et al. 2016; van der Ende et al. 2012). Differences between adult informants in ASD symptomatology have also been found (e.g., Baron-Cohen et al. 2001; Möricke et al. 2016). However, inter-rater correlations between self- and other ASD reports in adults have been considered satisfactory (e.g., Baron-Cohen et al. 2001; Noens et al. 2012) and agreement between self and others has been found to be moderate on social responsiveness (De la Marche et al. 2015).

The primary goal of the current cross-sectional study is to test the association between age and ASD characteristics, including empathy and sensory sensitivity, in adults aged 19–79 years. The current research set-up is highly similar to the recent Happé study (2016) as the majority of the participants have an adulthood diagnosis of ASD, have an (above) average intelligence, and the general ASD traits [i.e., autism-spectrum quotient (AQ)] measure included is exactly the same. Therefore, we expect to replicate the finding that older age is associated with increased ASD self-reports, at least in those aged between 19 and 55.^1^ Moreover, we hypothesize higher ratings of cognitive and affective empathy up to mid age followed by lower ratings in late adulthood (inverted U-shape) and no relationship between age and sensory sensitivity. Furthermore, the role of sex is explored given the often observed symptomatic differences between males and females (Baron-Cohen et al. 2014; Hull et al. 2017; Lai et al. 2011; Ruzich et al. 2015; but see, van Wijngaarden-Cremers et al. 2014).

The secondary goal of the current paper is to compare self- and other-reported ASD characteristics. We test whether age- and sex-related findings are differently reported by informants, as this could be hypothesized based on previous studies. Furthermore, in line a much smaller study with an adult sample (De la Marche et al. 2015), we expect the agreement between self- and other-reports to be moderate.

## Methods

### Participants

Individuals with ASD aged 19–79 years were recruited through several mental health institutions across the Netherlands and by means of advertisement on client organization websites. Requirement upon study participation was to have a clinical ASD diagnosis based on DSM-IV criteria (autism, Asperger’s syndrome, and Pervasive Developmental Disorder Not Otherwise Specified) (American Psychiatric Association 2000), which was generally established by a multidisciplinary team including a psychiatrist and/or psychologist. Individuals without ASD [comparison group (COM)] were recruited by means of advertisement on the university website and social media and within the social environment of the experimenters. Controls were eligible for participation when a clinical diagnosis of ASD, attention deficit hyperactivity disorder (ADHD), schizophrenia and close relatives suffering from ASD or schizophrenia were absent. Based on these criteria we excluded four individuals with ASD and nine individuals without ASD, resulting in a sample of 440 participants (241 ASD, 199 COM). See for a detailed participants description also the Lever and Geurts (2016b) paper focusing on comorbidity in adults with ASD as here we largely included the same participants.

Of this sample of 440 participants, 435 participants completed the AQ (n_ASD_ = 237, n_COM_ = 198), 349 the Interpersonal Reactivity Index (IRI) (n_ASD_ = 172, n_COM_ = 177) and 336 the sensory sensitivity questionnaire (SSQ) (n_ASD_ = 163, n_COM_ = 173). Two-hundred-eighty-five participants returned one or more questionnaires filled out by an informant (e.g., this could be a partner, family member, or friend). Please note that the number of other-SSQs is by definition smaller than both the AQs and IRIs due to the later addition of the SSQ to the set of questionnaires. In total, 270 AQs (n_ASD_ = 125, n_COM_ = 145), 278 IRIs (n_ASD_ = 130, n_COM_ = 148), and 141 SSQs (n_ASD_ = 65, n_COM_ = 76) were completed. In a subset of the sample, the Autism Diagnostic Observation Schedule module 4 (ADOS; de Bildt and de Jonge 2008; Lord et al. 2000) (n_ASD_ = 142) was administered to have more detailed information regarding current ASD related symptomatology, IQ was estimated with a short version of the Wechsler Adult Intelligence Scale third edition (Uterwijk 2000; Wechsler 1997) (n_ASD_ = 142, n_COM_ = 180), and comorbidity was measured with self-reports and diagnostic interviews (see Lever and Geurts 2016b for details). In this subset, eligible ASD individuals were selected based on age and sex to ascertain that participants were evenly distributed across ages and sex until a predefined number of participants needed was reached.

### Measures

#### Autism-Spectrum Quotient (AQ)

The Dutch version of the AQ (Baron-Cohen et al. 2001; Hoekstra et al. 2008; Ruzich et al. 2015; Woodbury-Smith et al. 2005) was administered to identify the degree to which an intellectually able adult show ASD traits.^2^ This self-report questionnaire consists of 50 statements about core ASD-related characteristics and assesses five different areas: social skills, attention switching, attention to detail, communication, and imagination. Each statement is rated with 1 “definitely agree”, 2 “slightly agree”, 3 “slightly disagree”, and 4 “definitely disagree”. On half of the items, endorsement of “definitely agree/slightly agree” is indicative of ASD-like behavior, whereas on the other half “definitely disagree/slightly disagree” reveals ASD traits. These latest scores are reversed. The item scores are summed, to a maximum score of 10 per subscale and a maximum total score of 50. The other-version omits 10 items as these were labeled by the developers as being too subjective to be answered by another person (Baron-Cohen et al. 2001). Higher scores indicate more severe ASD traits. The Dutch version of the AQ has good internal consistency, test–retest reliability, and good discriminative validity (Hoekstra et al. 2008). Missing data points (maximum one per subscale) were substituted with the mean subscale score. The dependent variables are the total and subscale scores (self-report) and 40-item total score (self- and other-report).

#### Interpersonal Reactivity Index (IRI)

The Dutch version of the IRI (Davis 1980; de Corte et al. 2007) is a widely-used instrument to examine individual differences in cognitive and emotional attitude towards interpersonal situations. This self-report questionnaire consists of 28 items and four subscales assessing different aspects of empathy, which is crucial of normal social functioning, including the maintenance of social relationships and favoring pro-social behavior (de Corte et al. 2007): (a) perspective taking, the tendency to adopt another person’s point of view, (b) fantasy, the tendency to identify with the feelings and actions of fictitious characters, (c) empathic concern, the tendency to experience feelings of sympathy and concern towards others, and (d) personal distress, the tendency to feel anxious and uneasy in reaction to the emotions of others (Davis 1983). The first two subscales examine other-oriented behavior (cognitive component), whereas the latter two subscales examine self-oriented behavior (affective component). Each item is rated on a five-point Likert scale, ranging from 0 “does not describe me well” to 4 “describes me very well”. The item scores are summed to a maximum of 28 per subscale. While higher perspective-taking scores and lower personal distress scores are associated with better social functioning, correlations between social functioning and fantasy are low. Empathic concern is not consistently related to social competence, although associated with social success characteristics, such as selflessness and agreeableness. The Dutch version of the IRI has adequate psychometric properties (de Corte et al. 2007). Missing data points (maximum one per subscale) were substituted with the mean subscale score. The dependent variables are the subscale scores (self-report) and total score (self- and other-report).

#### Sensory Sensitivity Questionnaire (SSQ)

The SSQ (Minshew and Hobson 2008) is, after permission of the authors, translated from English into Dutch (Lever and Geurts 2012) and back-transformed into English by an independent native English speaker. The SSQ consists of 13 statements about sensory hyper- or hyposensitivity that can be endorsed or denied, and assess low pain/temperature (2 items), high pain/temperature (2 items), tactile sensitivity (3 items), and other sensitivities (6 items). Endorsed items are summed per subscale and to a total score of maximum 13. Inter-rater reliability is good (Minshew and Hobson 2008), but other psychometric properties of the SSQ are yet unknown. Missing data points for SSQ were not allowed due to the small number of questionnaire items. The dependent variable is the total score (self- and other-report).

### Procedure

After explanation of study purposes and procedure, written informed consent was obtained for all participants. The AQ, IRI, and SSQ questionnaires were filled out. Additional questionnaires were filled out and additional measures were administered in two sessions in a selection of this sample, but these were described elsewhere (Lever and Geurts 2016a, b; Lever et al. 2015, 2017). The study was approved by the local institutional ethical review board (2011-PN-1952), and complied with all relevant laws and institutional guidelines.

### Statistical Analyses

First, to compare the ASD and COM group on descriptive measures, ANOVAs (continuous variables) and Fisher’s test (categorical variables) were used.

Second, to investigate age-related differences in ASD symptomatology, two MANCOVAs for AQ and IRI (sub)scales and an ANCOVA for the SSQ total score^3^ were run. Group and sex were the between-subject factors and age and age^2^ were included as covariates in a model with main effects and interactions. Sex was included as between-subject factor to explore the role of sex. Age and age^2^ were both centered to ease interpretation. Separate ANCOVAs on the single (sub)scales (Bonferroni correction: α = 0.05/6 = 0.0083 for AQ; α = 0.05/4 = 0.0125 for IRI) were used to follow-up on the omnibus MANOVA effects. When observing significant interactions, we ran planned follow-up regressions analyses (Bonferroni correction: α = 0.05/number of significant interactions) per group. Third, to examine the relation between self- and other-report, intra-class correlations coefficients (ICCs) were calculated with a two-way mixed, absolute agreement, single-measures effect model (Hallgren 2012; McGraw and Wong 1996; Shrout and Fleiss 1979), overall and per group, for total scores of AQ (40 items), IRI (all items), and SSQ (all items). Levels of agreement were interpreted as poor (ICC < 0.40), fair (ICC = 0.40–0.59), good (ICC = 0.60–0.74), and excellent (ICC = 0.75–1.00) (Cicchetti 1994). To further examine the self-other relationship, we computed three ANOVAs with Group (ASD, COM) as between-subject factors and Rater (self, other) as within-subject factor. Furthermore, to examine whether age-related differences were also observed by proxies (i.e., other-report), we ran ANCOVAs for each questionnaire’s total score, with group and sex as between-subject factors and centered age and centered age^2^ as covariates. Additional exploratory analyses are described in the Online Resources 1, 2, and 3. All analyses were run with SPSS 22.0 (IBM Corp. 2013).

## Results

**Fig. 1:**
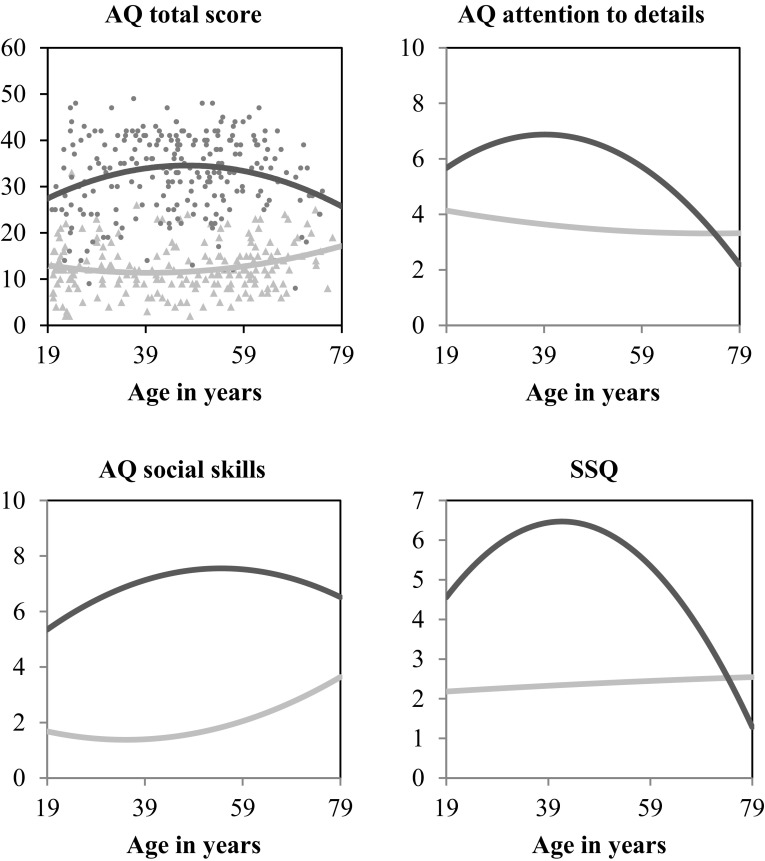
Age-related differences on the AQ total score, AQ social skills subscale, AQ attention to detail subscale, and SSQ. The darker line and dots indicate the group with autism spectrum disorder

The descriptives of both groups (i.e., sex, age, social characteristics, years of diagnosis) are depicted in Table 1.^4^ The age distribution is shown in Fig. 1, AQ total score (79 individuals with ASD between 19 and 40 years completed the AQ, 79 between 40 and 53 years, and 79 between 53 and 79 years). The groups did not differ in mean age, but the ASD group was composed of relatively more males than females as compared to the COM group. Self-reported questionnaire scores of the ASD and COM group and subscale comparisons are presented in Table 2. Follow-up regressions on significant interactions between age(^2^) and group are presented in Table 3.

**Table 1:** Comparisons of descriptive variables

	ASD (n = 237)	COM (n = 198)	Statistics
Age (years)	46.0 (SD 13.8) Range 19–79	45.6 (16.4) Range 19–77	*F*(1, 433) = 0.08, *p* = .773, *η*_p_^2^ = 0.00
Sex	163 M/74 F	109 M/89 F	Fisher’s test, *p* = .004, odds ratio = 1.80
Educational level^a^			Fisher’s test, *p* < .001
Low	1	0	
Middle	86	41	
High	147	156	
Residential status			Fisher’s test, *p* < .001
Independent	97	64	
With partner or housemate	107	116	
With parents	13	17	
Residential home	19	0	
Other	1	1	
Relationships			Fisher’s test, *p* = .019
Unmarried	106	71	
Married	87	88	
Cohabiting	21	29	
Other	23	10	
Diagnosis		–	–
Autistic disorder	42		
Asperger syndrome	117		
PDD-NOS	71		
ASD	7		
Time of diagnosis (years)	4.0 (3.9) Range 0–26	–	–
Occupation^a^			χ^2^ = 8.82, *p* = .032
Unemployed^b^	95	68	
Class 1–3	87	90	
Class 4–6	30	24	
Class 7–9	19	5	
SCL-90 (mean)^c^	174.8 (52.7)	113.5 (22.4)	*F*(1, 348) = 202.4, *p* < .001, *η*_p_^2^ = 0.37
IQ (mean)^c^	113.1 (17.7)	112.6 (17.3)	*F*(1, 321) = 0.07, *p* = .790, *η*_p_^2^ = 0.00
ADOS (mean)^c^	8.7 (3.4)	–	–

*ASD* autism spectrum disorder, *COM* comparison group, *M* male, *F* female, *PDD-NOS* pervasive developmental disorder not otherwise specified, *ISCO* International Standard Classification of Occupations, *SCL-90* symptom checklist-90, *IQ* estimated intelligence quotient, *ADOS* autism diagnostic observation schedule

^a^Missing: educational level: 3 ASD, 1 COM; occupation: 6 ASD, 11 COM

^b^Unemployment also included retirement and students

^c^SCL-90, IQ, and ADOS were assessed in a subgroup of participants (ASD: IQ/ADOS n = 142, SCL-90 n = 172; COM: IQ n = 180, SCL-90 n = 177)

**Table 2:** Group comparisons of the self-reported questionnaires

	ASD	COM	Group		Sex		Group × sex	
	M (SD)	M (SD)	*F*	*η*_p_^2^	*F*	*η*_p_^2^	*F*	*η*_p_^2^
AQ								
Total score	32.9 (8.4)	12.5 (5.5)	560.86***	0.57	0.68	0.00	12.55***	0.03
Social skills	7.1 (2.5)	1.8 (1.9)	399.62***	0.48	0.06	0.00	8.05**	0.02
Attention switching	7.5 (2.2)	2.5 (1.8)	428.42***	0.50	0.78	0.00	8.98**	0.02
Attention to detail	6.2 (2.4)	3.6 (2.2)	110.03***	0.21	3.84	0.01	0.57	0.00
Communication	6.4 (2.4)	1.8 (1.5)	345.84***	0.45	0.04	0.00	9.66**	0.02
Imagination	5.7 (2.2)	2.8 (1.8)	128.07***	0.23	0.06	0.00	6.44*	0.02
IRI								
Perspective taking	12.6 (5.2)	18.3 (3.9)	86.58***	0.20	0.00	0.00	6.97**	0.02
Fantasy	12.5 (6.1)	14.9 (5.6)	6.25*	0.02	1.20	0.00	6.65*	0.02
Empathic concern	15.6 (4.9)	17.2 (4.3)	0.99	0.00	29.29***	0.08	1.81	0.01
Personal distress	14.9 (5.4)	10.1 (4.7)	53.29***	0.14	13.92***	0.04	0.51	0.00
SSQ								
Total	5.6 (2.9)	2.4 (1.9)	145.54***	0.31	27.22***	0.08	8.01**	0.02

	Age		Age^2^		Group × age		Group × age^2^	
	*F*	*η*_p_^2^	*F*	*η*_p_^2^	*F*	*η*_p_^2^	*F*	*η*_p_^2^
AQ								
Total score	3.51	0.01	2.48	0.01	12.92***	0.03	13.10***	0.03
Social skills	1.63	0.00	0.36	0.00	7.92**	0.02	7.36**	0.02
Attention switching	0.06	0.00	0.08	0.00	7.37**	0.02	7.49**	0.02
Attention to detail	3.62	0.01	6.20*	0.01	8.88**	0.02	10.50**	0.02
Communication	1.95	0.01	0.86	0.00	4.62*	0.01	4.20*	0.01
Imagination	2.19	0.01	0.83	0.00	2.23	0.01	2.18	0.01
IRI								
Perspective taking	0.63	0.00	0.89	0.00	1.20	0.00	0.79	0.00
Fantasy	0.85	0.00	0.18	0.00	0.15	0.00	0.23	0.00
Empathic concern	1.68	0.01	1.31	0.00	2.00	0.01	2.68	0.01
Personal distress	3.67	0.01	4.36*	0.01	0.59	0.00	0.40	0.00
SSQ								
Total	6.13*	0.02	7.02**	0.02	7.13**	0.02	9.02**	0.03

Significant values after Bonferroni correction (α= 0.05/6 = 0.0083 for AQ; α = 0.05/4 = 0.0125 for IRI) are indicated in bold script. Please note that no Bonferroni correction was needed for SSQ data

*ASD* autism spectrum disorder, *COM* comparison group, *AQ* autism-spectrum quotient, *IRI* interpersonal reactivity index, *SSQ* sensory sensitivity questionnaire

* *p* ≤ .05

** *p* < .01

*** *p* ≤ .001

**Table 3:** Regression analyses (regression analyses were run per group on the scales that yielded a significant interaction between group and age(^2^)) for effects of age on the self-reported questionnaires

	AQ total score		AQ social skills		AQ attention switching		AQ attention to detail		SSQ	
	ASD	COM	ASD	COM	ASD	COM	ASD	COM	ASD	COM
	*β*	*β*	*β*	*β*	*β*	*β*	*β*	*β*	*β*	*β*
Age	1.38***	− 0.89*	1.07**	− 0.73	0.86*	− 1.00*	1.34***	− 0.30	1.54***	0.08
Age^2^	− 1.36***	1.02*	− 0.92*	0.94*	− 0.92*	0.98*	− 1.60***	0.19	− 1.76***	− 0.02
Constant	34.54***	11.51***	7.43***	1.49***	7.80***	2.15***	6.73***	3.54***	6.39***	2.39***
R^2^	0.05	0.04	0.05	0.06	0.03	0.02	0.13	0.01	0.12	0.00
N	237	198	237	198	237	198	237	198	163	173

Significant values after Bonferroni correction (α = 0.05/5 = 0.01) are indicated in bold script

*ASD* autism spectrum disorder, *COM* comparison group, *AQ* autism-spectrum quotient, *SSQ* sensory sensitivity questionnaire

* *p* ≤ .05

** *p* ≤ .01

*** *p* ≤ .001

### Self-Reported ASD Characteristics: Group Differences

As expected, there was a significant main effect of group on the AQ (Wilks’ Lambda (Λ) = 0.40, *F*(5, 423) = 125.60, *p* < .001, *η*_p_^2^ = 0.60), IRI (Λ = 0.72, *F*(4, 338) = 32.86, *p* < .001, *η*_p_^2^ = 0.28), and SSQ (*F*(1, 335) = 145.54, *p* < .001, *η*_p_^2^ = 0.31). Adults with ASD reported higher scores on the SSQ and on all subscales of the AQ than adults without ASD. On the IRI, ASD adults reported lower scores on perspective taking and fantasy, comparable scores on empathic concern, and higher scores on personal distress.

### Self-Reported ASD Characteristics: Age

There was no significant effect between age or age^2^ and IRI scores. In contrast, AQ and SSQ scores were differently affected by age in the ASD and COM group as showed by significant interaction effects (AQ: group × age, Λ = 0.97, *F*(5, 423) = 3.02, *p* = .011, *η*_p_^2^ = 0.04, group × age^2^, Λ = 0.96, *F*(5, 423) = 3.21, *p* = .007, *η*_p_^2^ = 0.04; SSQ: group × age, *F*(1, 335) = 7.13, *p* = .008, *η*_p_^2^ = 0.02, group × age^2^, *F*(1, 335) = 9.02, *p* = .003, *η*_p_^2^ = 0.03). In the ASD group, increasing age was associated with higher scores on the AQ total score, AQ attention to detail, and SSQ. However, the effect of age^2^ indicated a peak of these traits in middle adulthood (Fig. 1). Furthermore, age was significantly associated with the AQ social skills subscale, with increasing age being related to higher scores without any peak. In the COM group, there was no relation between age(^2^) and any of the self-reported questionnaire scores.

### Self-Reported ASD Characteristics: Sex

Sex differences between the ASD and COM group were observed as shown by significant interaction effects (AQ: Λ = 0.97, *F*(5, 423) = 3.07, *p* = .010, *η*_p_^2^ = 0.04; IRI: Λ = 0.97, *F*(4, 338) = 2.83, *p* = .025, *η*_p_^2^ = 0.03; SSQ: *F*(1, 335) = 8.01, *p* = .005, *η*_p_^2^ = 0.02). ASD females reported higher scores than ASD males on the AQ total score (*β* = 0.19, *p* = .004), AQ attention switching subscale (*β* = 0.19, *p* = .004), and SSQ (*β* = 0.39, *p* < .001). IRI perspective taking and fantasy scores did not differ between females and males with ASD (respectively, *β* = − 0.11, *p* = .163 and *β* = − 0.05, *p* = .504). In contrast, non-ASD females reported lower scores than non-ASD males on the AQ total score (*β* = − 0.20, *p* = .006) and AQ communication subscale (*β* = − 0.23, *p* = .001), higher scores on the IRI perspective taking and fantasy subscales (respectively, *β* = 0.19, *p* = .010 and *β* = 0.21, *p* = .005), and no differences on the SSQ (*β* = 0.16, *p* = .039) after Bonferroni correction. Females reported higher scores on the IRI personal distress and empathic concern subscales than males in both groups (see Table 2).

### Differences Between Self- and Other-Reported ASD Symptoms

The informants were partners (55.0%), family members (28.4%), friends (11.3%), or others (2.8%), such as practitioners. Unfortunately, 2.5% did not indicate which type of relationship they had with the participant. Of two participants who handed in questionnaires of two different informants, we included data from one of these (i.e., the person who has known the participant for the longest time). The mean length of the relationship between participant and informant was 24.2 years (SD 13.2; median 24; range 0.5–57.0 years) and comparable between ASD and COM group (*p* > .4).

#### Informant Agreement

ICCs indicated fair (IRI, SSQ) to excellent (AQ) levels of agreement between self- and other-report for the total sample (see Table 4). Levels of agreement were fair for the COM group and poor to fair in the ASD group.^5^ Considering the 95% confidence intervals of each group, the levels of agreement differ between groups on the AQ, but not on the IRI and SSQ.

**Table 4:** Intra-class correlations, confident intervals, and self- and other-reported mean scores and standard deviations for each questionnaire

	N^a^	ICC	95% CI	Self M (SD)	Other M (SD)
Total					
AQ^b^	270	0.786***	0.724–0.834	17.9 (10.1)	19.7 (10.3)
IRI	275	0.476***	0.359–0.575	57.9 (13.4)	53.3 (14.8)
SSQ	134	0.534***	0.400–0.645	4.0 (3.0)	3.8 (2.6)
COM group					
AQ^b^	145	0.459***	0.315–0.581	10.1 (4.8)	11.7 (5.7)
IRI	146	0.471**	0.334–0.588	60.6 (13.1)	57.7 (13.5)
SSQ	72	0.473***	0.275–0.633	2.5 (2.0)	2.9 (2.2)
ASD group					
AQ^b^	125	0.187*	0.020–0.346	26.9 (6.5)	28.9 (5.4)
IRI	129	0.411***	0.225–0.561	54.7 (13.1)	48.4 (14.6)
SSQ	62	0.390***	0.163–0.580	5.6 (3.0)	4.9 (2.6)

*ASD* autism spectrum disorder, *COM* comparison group, *ICC* intra-class correlation coefficient, *CI* confidence interval, *AQ* autism-spectrum quotient, *IRI* interpersonal reactivity index, *SSQ* sensory sensitivity questionnaire

* *p* ≤ .05, ** *p* ≤ .01, *** *p* ≤ .001

^a^Please note that the numbers of participants included in the analyses are slightly lower than the numbers reported in the participant section as for these analyses only those individuals were included who had completed self- and other-report

^b^Please note that self-reported AQ scores are lower than in Table 2 as 10 items are omitted for the comparison with other-report (see “Methods” for details)

**Fig. 2:**
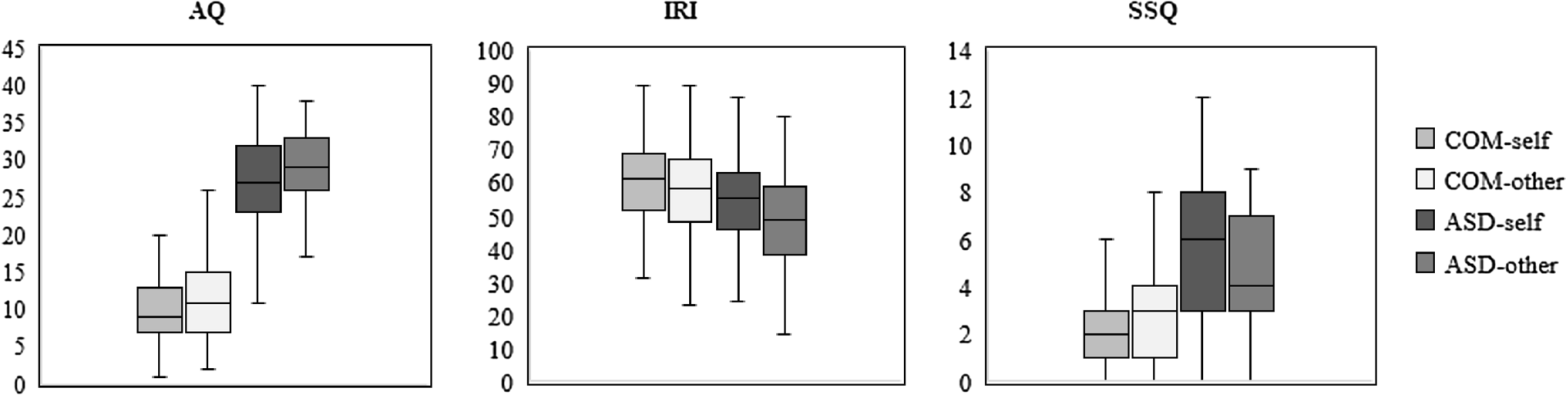
Box and whisker plots for self- and other-report on the Autism-Spectrum Quotient (AQ), Interpersonal Reactivity Index (IRI), and Sensory Sensitivity Questionnaire (SSQ) for both the comparison (COM) and autism spectrum (ASD) group

Comparison of self- and other-report revealed a main effect of rater on the AQ (*F*(1, 268) = 19.93, *p* < .001, *η*_p_^2^ = 0.07), with lower ratings for self-report than for other-report, but no interaction between rater and group (*F*(1, 268) = 0.36, *p* = .548, *η*_p_^2^ = 0.00) (Fig. 2). Hence, the differences between raters do not seem to be more pronounced in the ASD group. On the IRI, there was an interaction between rater and group (*F*(1, 273) = 4.09, *p* = .044, *η*_p_^2^ = 0.02). Proxies reported lower scores than participants themselves in both groups, but follow-up comparisons revealed that this discrepancy was more pronounced in the ASD group (ASD: *F*(1, 128) = 24.76, *p* < .001, *η*_p_^2^ = 0.16; COM: *F*(1, 145) = 6.82, *p* = .010, *η*_p_^2^ = 0.05). Rater and group also interacted on SSQ scores (*F*(1, 132) = 5.98, *p* = .016, *η*_p_^2^ = 0.04) with lower ratings for other-report than for self-report in the ASD group and vice versa in the COM group. Follow-up comparisons revealed that apparent differences were too small and variability too large to detect significant differences between self- and other-report in both groups (ASD: *F*(1, 61) = 3.27, *p* = .076, *η*_p_^2^ = 0.05; COM: *F*(1, 71) = 2.53, *p* = .116, *η*_p_^2^ = 0.03).^6^

#### Group, Age and Sex

Group differences were also revealed by other-reports (all *p*s ≤  .009, *η*_p_^2^ = 0.03–0.52). However, age-related differences as reported by proxies were not found to be significant on neither the AQ, IRI, nor SSQ (all *p*s >  .07). Moreover, proxies reported higher IRI (*p* < .001, *η*_p_^2^ = 0.08) and SSQ scores (*p* = .005, *η*_p_^2^ = 0.06) for females than for males, but, in contrast to the self-reports, similar AQ scores (*p* = .095, *η*_p_^2^ = 0.01). Please note that age- and sex-related findings were similar in both groups.

## Discussion

To our knowledge, this is the first study focusing on age-related differences in self- and other-reported ASD characteristics in a large sample of intellectually able individuals with clinical ASD across the adult lifespan including old age. The findings show clearly that age, sex, and type of informant are crucial to take into account when studying ASD characteristics across adulthood.

### Self-Report: Group-, Sex-, and Age-Related Differences

As expected, adults with ASD reported more ASD traits (e.g., Baron-Cohen et al. 2001; Ruzich et al. 2015), higher sensory sensitivity (Crane et al. 2009; Minshew and Hobson 2008), and lower perspective taking and fantasy tendencies, similar empathic concern, and higher personal distress in reaction to the emotions of others (Rogers et al. 2007) than individuals without ASD. Moreover, we replicated earlier findings that females with ASD had more sensory issues and reported more ASD characteristics than males (Happé et al. 2016; Lai et al. 2011), whereas females without ASD manifested fewer ASD traits than non-ASD males (Ruzich et al. 2015).

Within the ASD group, age-related differences were observed in self-reported ASD traits and sensory sensitivity, with a peak among middle-aged adults. These results are apparently in contrast with the few cross-sectional studies investigating the role of age on self-reported ASD symptoms in (younger) adults as these studies did not find any association with age (Bastiaansen et al. 2011; Bishop and Seltzer 2012; Crane et al. 2009; Minshew and Hobson 2008) or found more ASD traits associated with older age (Happé et al. 2016). However, these studies did not consider a non-linear relationship (Bastiaansen et al. 2011; Bishop and Seltzer 2012; Crane et al. 2009; Minshew and Hobson 2008), had a small sample size (Crane et al. 2009) or included only a few individuals aged over 55 (Happé et al. 2016). The high number of self-reported ASD characteristics in middle adulthood found in our study and the Happé study, suggests that ASD characteristics are more heavily experienced in middle adulthood than in younger or older adults. Not only ASD characteristics are most pronounced in middle adulthood, also comorbid psychopathology is frequently experienced in this life period (Lever and Geurts 2016b).^7^ Middle adulthood is associated with increased demands of responsibility, shifting roles, and adjustments to life changes. People may need to deal with changes in multiple domains, including psychosocial, emotional, and physical changes (Lachman 2004), that require substantial resources to adequately face them. These resources could be less efficient in individuals with ASD, causing distress and highlighting their ASD traits. Regarding sensory sensitivity, reduced sensory functioning (Fozard 1990) or better coping mechanisms (Grandin 2011) in older adulthood may additionally explain the fewer reported characteristics in old age.

In both adults with and without ASD, empathy, an aspect of social-emotional reciprocity, was not sensitive to age-related differences (e.g., Eysenck et al. 1985). It has previously been demonstrated that age-related differences in perspective taking and empathic concern may follow an inverted U-shape (O’Brien et al. 2013). However, this pattern was found in a very large sample of more than 75,000 individuals drawn from the general population. Our failure to replicate this finding is plausibly a power issue as the directions of estimated coefficients in the current study were comparable, even though our results fit ASD-related findings indicating that age did not affect cognitive reasoning on other persons’ mental states (Chung et al. 2014).

Within the comparison group, as in previous reports about the general population, age was not associated with general ASD symptoms (Hoekstra et al. 2008; but see; Broadbent et al. 2013) or sensory sensitivity (Crane et al. 2009; Robertson and Simmons 2013).

### Self- Versus Other-Report

Overall, the current results show poor to fair agreement between self- and other-reports of well-known proxies, even though the agreement of the overall group was similar to those previously reported for social responsiveness (De la Marche et al. 2015). Given that rather low agreement was observed in both the ASD and COM group, it seems unsuitable to conclude that this is due to poor metacognitive abilities in ASD, as has been previously argued (Frith 2004; Johnson et al. 2009; Kievit and Geurts 2011). Rather, a rater bias (De Los Reyes 2013; Hirschfeld 1993; John and Robins 1993; Leising et al. 2010) or a different way of perceiving or experiencing behavioral traits (Carlson et al. 2013) may reflect the discrepancy between self- and other-report. For example, a person may enhance one’s own characteristics (John and Robins 1993) or experience his or her so-called “pathological” traits as more acceptable or desirable than an informant (Hirschfeld 1993) and, hence, underestimate the degree of behavioral characteristics relative to others. Simultaneously, proxies may focus more on the so-called “pathological” traits than on, what they perceive as, typical traits (Leising et al. 2010) and, hence, overestimate certain symptoms. Or, the self may be more accurate about traits that describe unobservable thoughts and feelings due to privileged access (e.g. feelings of empathy and sensory sensitivity), whereas an informant would be more accurate about observable behavior (e.g., ASD traits) (Vazire 2010). Also, people can behave differently in different settings, so certain traits may not be visible to proxies (De Los Reyes 2013). To disentangle these different explanations is a potentially interesting future research avenue. Please note that the mean difference in AQ score between self and other (i.e., 1.8) was smaller than in the original Baron-Cohen sample (i.e., 2.8; 2001), which has been described as good, even though statistical analyses were lacking.

Regarding the presence of more self-reported ASD traits by females, which are not reported by proxies, it may support the idea that females are, in general, better in camouflaging (i.e., masking or compensating for) their condition (Dean et al. 2017; Lai et al. 2015, 2016; Rynkiewicz et al. 2016). This is in line with the finding that females showed less symptoms than males on a clinician-rated measure (i.e. the ADOS; Online Resource 3). Alternatively, females may more strongly perceive their symptoms or, although speculative, females may feel the need to report more ASD symptoms in order to be recognized as having ASD, getting access to the mental health system and receiving appropriate treatment, as ASD in girls and women is still underdiagnosed (Halladay et al. 2015). In sum, using self-report to gain insight into a person’s experience and understanding of certain feelings, thoughts, and behaviors should also be considered as a valuable tool for intellectually high functioning adults with ASD, while discrepancies between self- and other-report seem to capture different aspects of ASD characteristics.

### Limitations

Several limitations of this study should be acknowledged. ADOS and IQ were only assessed within a subgroup of the clinical sample. However, as demographics and self-reported scores did not differ between the subgroup and the entire sample (Online Resource 1), it is expected that the results extend to the overall ASD sample included in this study. As such, the sample consisted of intelligent individuals, with many having a paid job (some high profile), living with a partner, and being diagnosed with ASD relatively late in life.^8^ Therefore, the group is not representative of the entire autism population and the results cannot be generalized to the whole spectrum. However, the sample is representative of those receiving an adulthood diagnoses, which is a group which has previously been largely ignored within ASD research (but see Geurts and Jansen 2012; Happé et al. 2016), and those typically seen in general adult mental health care across parts of Europe. Furthermore, recent reports indicated that a majority of individuals with ASD may not present intellectual disability (Brugha et al. 2016; Christensen et al. 2016). As the ADOS was only administered to individuals with ASD, the information it provided regarding age, sex, and self- and other-reported questionnaire associations may be obscured by the lack of comparison with the control group. Also age, sex, and intelligence could confound interpretation of the ADOS module 4 (Bastiaansen et al. 2011; Hus and Lord 2014; Pugliese et al. 2015). Moreover, the cross-sectional nature of the study does not allow to draw conclusions on how self-reported ASD characteristics change over the years *within*-persons. A longitudinal follow-up is needed to investigate whether age-related changes in ASD symptoms, generally examined with measures relying on other information (i.e., a parent or caregiver, e.g. Howlin et al. 2013), are also detected by ASD individuals themselves and whether this change trajectory is indeed one of improvement. Finally, cohort effects could have occurred as a result of changes in social and cultural perspectives of ASD. Despite these limitations, to the current findings do have some potential clinical implications for this intelligent group of individuals.

### Clinical Implications

The age-related differences observed in self-reported ASD characteristics suggest that it would be meaningful to inquire after the experience of symptoms at different time-points within the adult lifespan instead of just assessing this at the time of diagnosis. Even though such repeated assessment might be challenging in some countries due to the (lack of) access to mental health care for ASD adults, it is of importance that ASD adults get the opportunity to have a regular checkup in order to provide individually tailored care which is age-appropriate. Moreover, the self-reported sex differences on ASD traits and sensory sensitivities underline that clinical professionals should be aware of symptomatic differences between males and females. Finally, our findings also suggest that it is important to rely on more than one source for diagnostic assessment (National Institute for Health and Clinical Excellence 2012; Trimbos 2013). Whether the other informant is a partner, family member, or friend may yield subtle differences in the amount of reported ASD characteristics. While friends reported less ASD traits and more empathy compared to partners, discrepancies between self- and other-report were the smallest for partners on the AQ, for friends on the IRI, and for family members on the SSQ (Online Resource 2). Associations between a clinician-rated measure of ASD symptomatology (i.e., the ADOS) and self- and other-reported questionnaires on ASD characteristics were also very weak (Online Resource 3). In general, as clients and proxies seem to perceive different aspects of ASD symptomatology, the discrepancies may provide an interesting contrast to discuss during assessment.

## Conclusions

In this large cross-sectional study of adults with clinical diagnoses of ASD, we demonstrated that adults with ASD experience a significant degree of ASD characteristics, empathic difficulties, and sensory sensitivities across adulthood. However, in line with the suggestion that ASD characteristics may fluctuate over the lifespan, age-related differences in ASD traits and sensory sensitivities were observed. Self-reported ASD traits and sensory sensitivities are highest in middle adulthood, and lower in young and older adulthood. Nevertheless, these age-related differences were not reported by proxies who have known the participants for a long time. Self and proxies may grasp distinct aspects of symptomatology. Longitudinal follow-up studies should reveal whether self-reported ASD symptoms are experienced to change over time.

## Electronic supplementary material

Below is the link to the electronic supplementary material.

Supplementary material 1 (PDF 13 KB)

Supplementary material 2 (PDF 58 KB)

Supplementary material 3 (PDF 56 KB)

## Footnotes

1 Please note that when we designed the study we actually expected a decrease in overall ASD symptomatology in the ASD group and not in the control group given (a) the previous findings using different measures that with increasing age there was a decrease in ASD symptomatology in those with ASD (Seltzer et al. 2004); and (b) that with the same measure in the general population no association with age was observed (Hoekstra et al. 2008). Our hypothesis changed due to the recent findings by Happé et al. (2016) and our own recent psychiatric comorbidity findings (Lever and Geurts 2016b).

2 When designing the study, no other Dutch self-reported measures for ASD traits or sensory sensitivity were available.

3 Data of the AQ subscales and SSQ total score were not normally distributed. However, as (M)ANOVA is thought to be robust against skewed data (Stevens 2009), we ran parametric tests.

4 We cross-checked whether the whole sample differed from the IRI or SSQ subsample on age, sex, and educational level. The groups did not significantly differ (all *p*s > .5).

5 ICCs for the whole group are typically larger than ICCs for subgroups.

6 To check whether the length of the relationship between participant and informant affected the results, these analyses were rerun with length of relationship as covariate. It did not alter the pattern of findings, except that the interaction between rater and group on the IRI was not significant anymore (*p* = .079).

7 Given that middle adulthood is also a crucial period for comorbid psychopathology, we exploratively verified whether the amount of self-reported psychological distress as measured with the Symptom Checklist-90 (SCL-90) (Arrindell and Ettema 2005; Derogatis 1977) could explain the peak in self-reported ASD characteristics as measured with the AQ and SSQ. It could not, as the age effects remained after correcting for SCL-90 scores.

8 We verified whether the time of diagnosis was related to the amount of self-reported ASD characteristics. AQ total, AQ subscale and SSQ scores were negatively correlated to time of diagnosis (*p*’s between − .147 and − .285, all p’s ≤ .029), but not to IRI scores (all *p*’s ≥ .195). However, associations were weak (Cohen 1988) and time of diagnosis did not affect the main findings.
