# Supplementary material for: Is Older Age Associated with Higher Self- and Other-Rated ASD Characteristics?
Source: J Autism Dev Disord. 2018 Jan 18;48(6):2038–51. doi: 10.1007/s10803-017-3444-2 (PMC5948271; doi:10.1007/s10803-017-3444-2)
Supplement: Supplementary file 1 — Supplementary material 1 (PDF 13 KB) [file 10803_2017_3444_MOESM1_ESM.pdf]

Online Resources

Lever, A.G., Geurts, H.M. *Is older age associated with higher self- and other-rated ASD characteristics?* Journal of Autism and Developmental Disorders.

Online Resource 1. Comparison non-ADOS, ADOS-, ADOS+, and ADOS++

Corresponding address

Dr. Hilde M. Geurts, Dutch Autism & ADHD Research Center, Department of Psychology, University of Amsterdam, Nieuwe Achtergracht 129B, 1018 WS Amsterdam, The Netherlands, e: H.M..Geurts@uva.nl, p: +31 20 525 6843, f: +31 20 639 1656.

Online Resource 1. Comparison non-ADOS, ADOS-, ADOS+, and ADOS++

Statistical analyses

Although all autism spectrum disorder (ASD) participants had a prior ASD diagnosis, these diagnoses were verified in a subgroup of participants (n=142) who were eligible to participate in a study aimed at investigating age-related differences in cognition (Lever & Geurts, 2016a) by administering the Autism Diagnostic Observation Schedule module 4 (ADOS; de Bildt & de Jonge, 2008; Lord et al., 2000). Therefore, we compared ASD participants who scored above the ADOS threshold for ASD (ADOS+; n=52) or autism (ADOS++; n=53) with those scoring below the threshold for ASD (ADOS-; n=37) or without ADOS (non-ADOS; n=95). The analyses were run with SPSS 22.0 (IBM Corp., 2013).

Results

The four ADOS groups did not differ in their mean age (p = .124), sex ratio (p = .246), educational level (p = .370), time of diagnosis (p = .841*)*, Autism-spectrum Quotient scores (p = .457), Interpersonal Reactivity Index scores (p = .351), or Sensory Sensitivity Questionnaire scores (p = .347). Hence, demographics and the amount of ASD characteristics did not differ between participants to whom the ADOS was not administered, to those scoring below the ASD threshold, and to those scoring above the ASD or autism threshold, suggesting that the results extend to the overall ASD sample.

References

de Bildt, A., & de Jonge, M. V. (2008). *Autisme Diagnostisch Observatie Schema.* Amsterdam: Hogrefe.

IBM Corp. (2013). *IBM SPSS statistics for Windows* (Version 22.0 ed.). Armonk, NY: IBM Corp.

Lord, C., Risi, S., Lambrecht, L., Cook Jr, E. H., Leventhal, B. L., DiLavore, P. C., . . . Rutter, M. (2000). The Autism Diagnostic Observation Schedule-Generic: A standard measure of social and communication deficits associated with the spectrum of autism. *Journal of Autism and Developmental Disorders, 30*(3), 205-223.
