# Supplementary material for: Is Older Age Associated with Higher Self- and Other-Rated ASD Characteristics?
Source: J Autism Dev Disord. 2018 Jan 18;48(6):2038–51. doi: 10.1007/s10803-017-3444-2 (PMC5948271; doi:10.1007/s10803-017-3444-2)
Supplement: Supplementary file 2 — Supplementary material 2 (PDF 58 KB) [file 10803_2017_3444_MOESM2_ESM.pdf]

Online Resources

Lever, A.G., Geurts, H.M. *Is older age associated with higher self- and other-rated ASD characteristics?* Journal of Autism and Developmental Disorders.

Online Resource 2. Effect of proxy type on ASD characteristics

Corresponding address

Dr. Hilde M. Geurts, Dutch Autism & ADHD Research Center, Department of Psychology, University of Amsterdam, Nieuwe Achtergracht 129B, 1018 WS Amsterdam, The Netherlands, e: H.M..Geurts@uva.nl, p: +31 20 525 6843, f: +31 20 639 1656.

Online Resource 2. Effect of proxy type on ASD characteristics

Statistical analysis

To explore whether the type of proxy influenced the number of reported ASD characteristics, we ran three exploratory ANOVAs with group (autism spectrum disorder [ASD], comparison [COM]) and type of proxy (partner, family, friend, or other [due to only a few cases, we clustered other proxies and unknown proxies together]) as between-subject factors for the total scores of the Autism-Spectrum Quotient (AQ), Interpersonal Reactivity Index (IRI), and Sensory Sensitivity Questionnaire (SSQ). The analyses were run with SPSS 22.0 (IBM Corp., 2013).

Results

Explorations on whether the type of proxy affected the amount of reported ASD characteristics, indicated a main effect on AQ and IRI (see eTable 1). Friends reported lower AQ scores than partners ($p = .001$), and others ($p = .010$), but not of family members ($p = .084$). On a similar note, friends reported higher IRI scores than partners ($p = .003$), even though the comparison with family members ($p =.065$) and others was not significant ($p = 1.000$). The other comparisons were also not significant. Hence, AQ and IRI proxy scores were influenced by who filled out the questionnaire. We explored which group of proxies diverged the most from the participants. In absence of an interaction between other-type and group, we combined the ASD and COM group. The discrepancies between self- and other-report were the smallest for partners on the AQ, for friends on the IRI, and for family members on the SSQ.

eTable 1. Means (standard deviations) per questionnaire for each proxy type, and statistics to compare scores of the ASD and COM group and the proxy type.

		Partner	Family member	Friend	Other
Total	AQ	20.3 (10.1)	19.8 (10.3)	14.0 (9.0)	24.8 (11.2)
	IRI	51.6 (15.1)	53.1 (15.1)	61.1 (9.1)	56.3 (15.4)
	SSQ	4.1 (2.5)	3.6 (2.6)	3.2 (2.8)	4.1 (2.3)
COM group	AQ	12.6 (5.7)	11.0 (5.5)	8.6 (4.1)	13.4 (9.7)
	IRI	56.9 (14.6)	57.6 (13.1)	61.4 (10.4)	57.2 (10.0)
	SSQ	3.5 (2.3)	2.0 (1.8)	1.7 (1.0)	3.0 (-)
ASD group	AQ	29.5 (5.5)	28.5 (5.1)	24.8 (5.5)	32.0 (3.3)
	IRI	45.3 (13.1)	48.7 (15.8)	60.5 (6.3)	55.8 (18.0)
	SSQ	4.9 (2.7)	4.8 (2.6)	5.5 (3.0)	4.3 (2.4)

	Statistics								
	Group			Proxy type			Group by proxy type		
	F	*p*	η_p^2	*F*	*p*	η_p^2	*F*	*p*	η_p^2
AQ	289.81	**<.001**	.53	5.88	**.001**	.06	0.20	.894	.00
IRI	5.27	**.022**	.02	4.42	**.005**	.05	1.60	.191	.02
SSQ	9.89	**.002**	.07	1.11	.349	.03	1.47	.227	.03

Note. ASD=autism spectrum disorder; COM=comparison group; AQ=Autism-Spectrum Quotient; IRI=Interpersonal Reactivity Index; SSQ=Sensory Sensitivity Questionnaire. Significant values are indicated in bold script.

References

IBM Corp. (2013). *IBM SPSS statistics for Windows* (Version 22.0 ed.). Armonk, NY: IBM Corp.
