# Supplementary material for: Is Older Age Associated with Higher Self- and Other-Rated ASD Characteristics?
Source: J Autism Dev Disord. 2018 Jan 18;48(6):2038–51. doi: 10.1007/s10803-017-3444-2 (PMC5948271; doi:10.1007/s10803-017-3444-2)
Supplement: Supplementary file 3 — Supplementary material 3 (PDF 56 KB) [file 10803_2017_3444_MOESM3_ESM.pdf]

Online Resources

Lever, A.G., Geurts, H.M. *Is older age associated with higher self- and other-rated ASD characteristics?* Journal of Autism and Developmental Disorders.

Online Resource 3. Clinician-rated ASD symptomatology (only ASD group)

Corresponding address

Dr. Hilde M. Geurts, Dutch Autism & ADHD Research Center, Department of Psychology, University of Amsterdam, Nieuwe Achtergracht 129B, 1018 WS Amsterdam, The Netherlands, e: H.M..Geurts@uva.nl, p: +31 20 525 6843, f: +31 20 639 1656.

Online Resource 3. Clinician-rated ASD symptomatology (only ASD group)

Statistical analyses

To exploratively examine the relationship between a clinician-rated symptomatology measure (i.e., the Autism Diagnostic Observation Schedule module 4 [ADOS; de Bildt & de Jonge, 2008; Lord et al., 2000]) and sex and age, we ran an ANCOVA with ADOS score (communication plus social domain score) as dependent variable, sex as a between-subject factor and age and age^2 as covariates in a subgroup of the ASD group (n = 142). Correlational analyses were also performed to explore the association between the ADOS and the self- and other-rated measures. The analyses were run with SPSS 22.0 (IBM Corp., 2013).

Results

There was no significant effect between age or age^2 and ADOS scores (age: $F(1, 138) = 2.25$, $p = .136$, $\eta_p^2 = .02$; age^2: $F(1, 138) = 1.07$, $p = .302$, $\eta_p^2 = .01$). Sex differences on ADOS scores were observed ($F(1, 138) = 7.81$, $p = .006$, $\eta_p^2 = .05$) with males showing higher scores (mean = 9.14, SD = 3.37) than females (mean = 7.68, SD = 2.82). Correlations between ADOS scores and self- and other-reported measures indicated very weak associations either when (see Table 1) or when not controlling for age (see Table 2). Please note that when controlling for age, the association between ADOS and IRI-self and IRI-other became negative, while the direction of the other associations did not change.

Table 1. Partial correlations between ADOS score and self- and other reported questionnaire scores (controlled for age).

		AQ-self	IRI-self	SSQ-self	AQ-other	IRI-other	SSQ-other
ADOS	Pearson's r	.039	-.120	-.043	.076	-.063	-.204
	p-value	.650	.162	.626	.438	.508	.165

Note. ADOS=Autism Diagnostic Observation Schedule module 4; AQ=Autism-Spectrum Quotient; IRI=Interpersonal Reactivity Index; SSQ=Sensory Sensitivity Questionnaire.

Table 2. Correlations between ADOS score and self- and other reported questionnaire scores.

		AQ-self	IRI-self	SSQ-self	AQ-other	IRI-other	SSQ-other
ADOS	Spearman's rho	.039	.026	-.102	.023	.064	-.178
	p-value	.806	.869	.514	.885	.682	.253

Note. ADOS=Autism Diagnostic Observation Schedule module 4; AQ=Autism-Spectrum Quotient; IRI=Interpersonal Reactivity Index; SSQ=Sensory Sensitivity Questionnaire.

References

de Bildt, A., & de Jonge, M. V. (2008). *Autisme Diagnostisch Observatie Schema.* Amsterdam: Hogrefe.

IBM Corp. (2013). *IBM SPSS statistics for Windows* (Version 22.0 ed.). Armonk, NY: IBM Corp.

Lord, C., Risi, S., Lambrecht, L., Cook Jr, E. H., Leventhal, B. L., DiLavore, P. C., . . . Rutter, M. (2000). The Autism Diagnostic Observation Schedule-Generic: A standard measure of social and communication deficits associated with the spectrum of autism. *Journal of Autism and Developmental Disorders, 30*(3), 205-223.
